# Translational Science in Ophthalmology

**Published:** 2012-01

**Authors:** Barry E Knox

**Affiliations:** SUNY Eye Institute, Departments of Neuroscience and Physiology and Ophthalmology, SUNY Upstate Medical University, Syracuse, NY, USA

Over the past decade, a new discipline in biomedical research has emerged. Translational science, as it has been termed, is concerned with the application of laboratory or “bench” science to the diagnosis and treatment of human diseases. The importance and potential impact of this new discipline for collaboration, education, funding and public interest have been widely discussed.^1^ Moreover; the role of well-informed clinicians, invested in promoting as well as utilizing new research, is clearly recognized as crucial for the communal advancement of medicine and continued support of basic science.^2^ To meet these emergent objectives for vision scientists, Journal of Ophthalmic and Vision Research is establishing a new section entitled *Translational Eye Research*, to stimulate communication between basic scientists and clinicians.

Basic research is expanding scientific knowledge at a phenomenal rate through *in vitro* experiments, investigations of model organisms and using cross-discipline methodologies. Yet, the development of improved clinical applications utilizing this information is complex and unpredictable. Progress does occur via studies designed to understand a specific disease or treatments developed according to defined strategies. Often, however, novel concepts arise in ways not readily connected to an obvious clinical problem. The language and ideas, not to mention the methods of investigation, can be foreign and impenetrable to those not yet experienced with the diverse experimental and biological systems in use today. The fact that many breakthroughs occur outside the realms of visual system research presents a severe and mounting challenge for ophthalmologists. This new section will meet the needs for the vision community by presenting up to date reviews in translational science. The articles will have two goals: first, to review an area of basic research at a level accessible to clinicians and second, to highlight clinical applications in ophthalmology. The ultimate goal is to present concepts and methods that may have applications not yet realized for treating patients. We hope to draw attention to basic science areas with potential for further development or exploration in the eye and ocular diseases. The goal is to stimulate the clinical audience to investigate unexpected avenues for possible applications in their own specialty and to expose basic researchers to opportunities for applying their expertise to clinical problems.
